# A Ni-based catalyst with polyvinyl pyrrolidone as a dispersant supported in a pretreated fluid catalytic cracking catalyst residue for C9 petroleum resin (C9 PR) hydrogenation

**DOI:** 10.1098/rsos.172052

**Published:** 2018-05-23

**Authors:** Dong Chen, Linlin Wang, Xiaopeng Chen, Xiaojie Wei, Jiezhen Liang, Jiao Jiang, Baofang Liang

**Affiliations:** 1 School of Chemistry and Chemical Engineering, Guangxi University, Nanning 53004, People's Republic of China; 2 Guangxi Key Laboratory of Petrochemical Resources Processing and Process Intensification Technology, Guangxi University, Nanning 53004, People's Republic of China

**Keywords:** hydrogenation, Ni catalyst, C9 petroleum resin, polyvinyl pyrrolidone

## Abstract

A Ni-based catalyst (Ni-PVP/PFC3R) with polyvinyl pyrrolidone (PVP) as a dispersant supported in a pretreated fluid catalytic cracking catalyst residue (PFC3R) was synthesized and applied to C9 petroleum resin (C9 PR) hydrogenation. For comparison, a Ni catalyst without PVP (Ni/PFC3R) was prepared in the same way. Ni-PVP/PFC3R exhibited higher activity and better stability. The catalysts were characterized by X-ray diffraction, scanning electron microscope, H_2_-temperature programmed reduction/temperature programmed desorption, Fourier transform infrared spectroscopy and the Brunauer–Emmett–Teller method. The catalysts had a smaller crystallite size and stronger interactions between the Ni species and the PFC3R support in the presence of PVP. The effects of nickel loading, H_2_ pressure, temperature and reaction time for C9 PR hydrogenation over Ni-PVP/PFC3R were investigated. The bromine number was reduced to 1.25 under the following conditions: nickel content of 12 wt%, PVP amount of 1.5 wt%, temperature of 270°C, H_2_ pressure of 8 MPa and reaction time of 240 min.

## 1. Introduction

**Figure 1.**
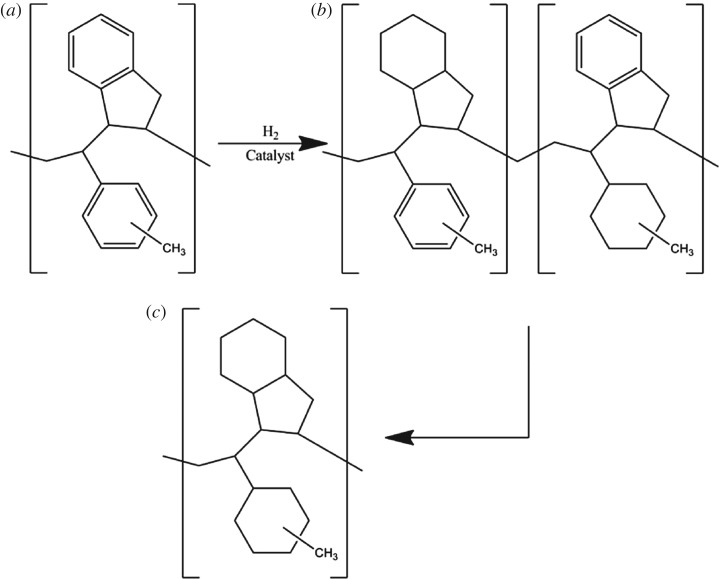
Scheme of C9 PR hydrogenation modification (*a*) starting material C9 PR; (*b*) partial hydrogenation; (*c*) complete hydrogenation.

C9 petroleum resin (C9 PR) is a thermoplastic polymer made from the polymerization of the C9 fraction (mainly vinyl toluene and indene, which are by-products from the catalytic cracking of petroleum hydrocarbons at high temperature during the preparation of ethylene) and has a molecular weight of 200 to 3000 and a softening point of 50–150°C [1]. It has a very wide range of applications in adhesives, paints, printing inks, coatings, rubber and other industries because of its excellent water resistance, acid and alkali corrosion resistance, weather resistance, light ageing resistance, miscibility and adhesion. C9 PR contains a lot of unsaturated hydrocarbons, such as dienes and aryl olefins. Therefore, C9 PR that has not been processed is usually yellow or amber and displays some shortcomings such as malodour, poor thermal stability, poor oxidation resistance, poor adhesion and poor compatibility [2]. The applications of C9 PR are limited. Hydrogenation can be used to modify C9 PR for wider industrial applications. A simplified structure of C9 hydrocarbon petroleum resin (C9 HPR) that has been used to explain the hydrogenation reaction in repeating units proposed by Kim *et al*. [3] is shown in figure 1. It has been reported that palladium-based or platinum-based catalysts show good catalytic properties in catalytic hydrogenation reactions [4–6]. However, noble metallic catalysts have common disadvantages, such as high cost, scarcity and poor tolerance. In recent years, nickel supported on alumina or carbon has received increasing attention because of their availability and low cost [7,8]. There have been some reports on multi-metal catalysts for resin hydrogenation [9–11]. However, few publications have reported the use of non-metallic additives for catalysts in this field.

Fluid catalytic cracking catalyst residue (FC3R) is the spent catalyst from the process in petroleum refining owing to coke and substantial deposits of heavy metal contaminants such as Ni, Fe and V [12]. Every year, over 300 000 tons of this solid waste [13], which mainly consists of SiO_2_ and Al_2_O_3_ [14,15], is discharged from petroleum refineries. It is mainly disposed in landfills, which not only pollutes the soil and groundwater, but also causes a high level of resource waste. Conditions for chemical and magnetic separation methods to remove heavy metals are very harsh; in those processes, the environment is subjected to secondary pollution because of the use of toxic gases, corrosive gases and acidic liquids [16–19]. Therefore, the reuse of FC3R is a very worthy research subject. FC3R possesses a well-developed specific surface and excellent physical strength, and it has great potential to be used as a catalyst carrier.

Polyvinyl pyrrolidone (PVP) has received special attention because of its high chemical stability, non-toxicity and excellent solubility in many polar solvents. PVP-stabilized metals not only exhibit a high degree of metal dispersion but are also stabilized against agglomeration [20–23]. Carotenuto *et al*. [24] and Wang *et al*. [25] have employed PVP as a protective agent to synthesize well-dispersed silver nanoparticles by a chemical reduction method; they found that PVP compounded with Ag and protected the silver particles from growing and agglomerating. Wan *et al*. [26] have prepared a polymer-supported palladium–manganese bimetallic catalyst for the oxidative carbonylation of amines to carbamate esters in the presence of PVP. The presence of PVP gives rise to an obvious increase in the conversion and selectivity of the reaction. Narayanan & El-Sayed [20] reported that the addition of excess PVP stabilizer to the reaction mixture may cause the inhibition of the Ostwald ripening process, resulting in the stability of the nanoparticle surface and size. Umegaki *et al*. [27] have synthesized a PVP-stabilized amorphous nickel catalyst by reduction in an aqueous NaBH_4_/NH_3_BH_3_ solution; they suggested that PVP prevented the agglomeration and crystallization of nickel nanoparticles, leading to higher durability than the bare nickel catalyst. However, few publications have reported the use of PVP as a dispersant or protective agent for supported catalysts, especially hydrogenation catalysts.

In this work, a waste-based material and economical Ni catalyst was prepared by an incipient wetness impregnation method using pretreated FC3R (PFC3R) as support and PVP as a dispersant and successfully used in C9 PR hydrogenation. For comparison, a Ni catalyst without PVP was prepared in the same way. The structures and properties of the supported Ni catalysts were characterized by X-ray diffraction (XRD), scanning electron microscope (SEM), X-ray photoelectron spectroscopy (XPS), Fourier transform infrared spectroscopy (FTIR), H_2_-temperature programmed reduction/temperature programmed desorption (H_2_-TPR/TPD) and the Brunauer–Emmett–Teller method (BET) and their catalytic performance in the C9 PR hydrogenation was extensively investigated.

## 2. Material and methods

### 2.1. Materials

Analytical-reagent grade Ni(NO_3_)_2_ 6H_2_O was purchased from Shanghai Xingao Chemical Reagent Co., Ltd (China), FC3R was provided by PetroChina Guangxi Tiandong Petrochemical Co., Ltd, as a coarse, porous and spherical material with a large BET surface area. The elemental composition of FC3R was characterized by SEM-energy-dispersive X-ray (EDX) and is shown in table 1. The PFC3R was obtained as follows: FC3R was calcined at 500°C to combust deposited coke and then immersed in 10% dilute sulfuric acid at 90°C for 6 h, rinsed 10–12 times with deionized water until the pH of the FC3R reached 7, and dried at 110°C for 4 h. The C9 PR was obtained from Lanzhou Yahua Petrochemical Co., Ltd., no. 200 industrial grade solvent oil (200^#^ solvent oil, boiling range is 140–200°C, aromatics content is 5 wt%) was purchased from Daming Jusen Chemical Co., Ltd., China, and was used as a solvent to dissolve the resin. Activated clay was purchased from Tianjin Guangfu Fine Chemical Research Institute, China, and was used as a sorbent to adsorb the impurities (mainly gel) in the raw material.

**Table 1.** Elemental analysis of FC3R using SEM-EDX (unit: %).

element	C	O	Na	Mg	Al	Si	S	Ca	Fe	Ni
content (%)	13.50	41.87	0.33	0.27	19.72	16.91	1.34	3.43	1.68	0.95

### 2.2. Preparation of catalyst samples

The PFC3R was impregnated by incipient wetness with a specific amount of an aqueous solution of Ni(NO_3_)_2_ 6H_2_O (Ni loading of 0.98, 4, 8, 12 or 16 wt%) and PVP (0, 0.5, 1.0, 1.5 or 3.0 wt%). The mixtures were stirred for 5 min at room temperature and then placed for 1 h under ultrasonication. After that, the sample was dried at 110°C for 4 h and the NiO-PVP/PFC3R was obtained by calcining at 450°C for 4 h in air (heating rate of 20°C min^−1^). Finally, the material was reduced under a flow of H_2_/N_2_ (flow rate of 30 ml min^−1^) at 450°C for 2 h to obtain Ni-PVP/PFC3R. For comparison, Ni/PFC3R was prepared by the same method only without adding PVP.

### 2.3. Analysis and characterization

**Figure 2.**
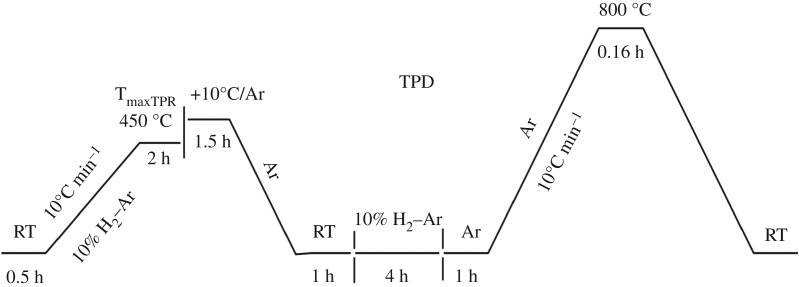
Schematic of temperature programmed desorption (TPD) methods employed in this study.

The SEM micrographs and the elemental composition of the catalysts were obtained with a Hitachi S4800 SEM that was equipped with an EDX detector. The XRD data were collected on a BRUCKER D8 within the range of 10 to 75° (2*θ*) operating at a scanning rate of 6° min^−1^ using Cu K*α* (*λ *= 1.541 Å) radiation. FTIR spectra were recorded with a Thermo scientific Nicolet 6700 FTIR spectrometer (4000–400 cm^−1^, KBr sheet). The Ni loading of the catalysts was determined by inductively coupled plasma–atomic emission spectrometry (ICP–AES) on a Perkin Elmer Optima 8000 spectrometer. The nitrogen BET surface area measurements were determined by a Micromeritics Instrument Corp Asap 2460 at −204°C. The XPS spectra were recorded with a Thermo Scientific Escalab 250Xi spectrometer. The TPR and H_2_-TPD experiments were carried out in a Micromeritics Chemisorb 2720 instrument. Approximately 300 mg of NiO/PFC3R was loaded into in a quartz reactor and heated at 300°C for 2 h in argon to remove the physisorbed molecules. The TPR was conducted with a temperature ramp rate of 10°C min^−1^ up to 900°C in a stream of 10% H_2_ in Ar, with a total flow rate of 30 ml min^−1^. The outlet gas was passed through a cold trap to remove moisture produced during the reduction. The TPD was carried out in a stream of argon with a flow rate of 30 ml min^−1^ and a heating ramp of 10°C min^−1^ up to 800°C (figure 2). Hydrogen consumption owing to the reduction of NiO was monitored using a thermal conductivity detector (TCD) linked to a computer data acquisition system. Ag_2_O was used as a standard to calibrate the TCD signals. The Ni metal surface area, dispersion and crystallite size were calculated from the volume of H_2_ chemisorbed by using the following simplified equations [28]:

Ni metal-specific surface area:

2.1 $${\mathrm{SA}}_{\mathrm{Ni}}({\text{m}}^{2}\;{\text{g}}^{-1}-\text{Ni})\;=\frac{V_{\mathrm{ad}}}{W_{S}}\times \frac{\text{SF}}{V_{m}}\times \frac{N}{F_{\mathrm{Ni}}}\times \mathrm{RA},$$

Ni dispersion (*D*):

2.2 $$D=\frac{V_{\mathrm{ad}}}{W_{S}}\times \frac{\text{F}{\text{W}}_{\mathrm{Ni}}}{F_{\mathrm{Ni}}}\times \frac{\text{SF}}{V_{m}}\times 100,$$

in which *V*_ad_* *= volume (ml) of H_2_ chemisorbed at standard temperature and pressure (STP) conditions to form a monolayer, *W*_s_ = weight of the sample (g), *V*_m_* *= molar volume of H_2_ (22 414 ml mol^−1^), SF = stoichiometric factor (Ni/H molar ratio during chemisorption), which is taken as 1, *N* = 6.023 × 10^23^ Ni atoms mol^−1^, RA = atomic cross-sectional area of Ni (0.0649 nm^2^), *F*_Ni_ = weight fraction of Ni in the sample as determined by ICP and FW_Ni_ = formula weight of Ni (58.71 g mol^−1^).

### 2.4. Catalyst performance tests

**Figure 3.**
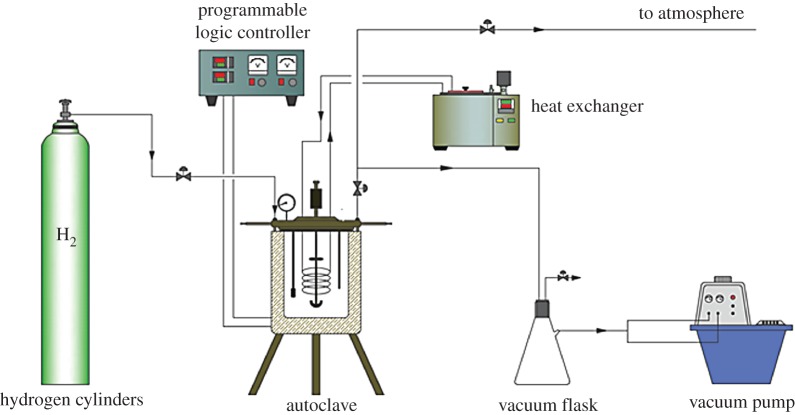
Scheme of the experimental set-up for C9 PR hydrogenation.

Catalytic hydrogenation experiments of C9 PR were carried out in a batch reactor, which was a 2 dm^3^ stainless steel autoclave (Dalian Jingyi Autoclave Co., Ltd., China). A schematic of the experimental set-up is depicted in figure 3. Approximately 900 g of solution pretreated with the activated clay of C9 PR and 200^#^ solvent oil (the mass ratio was 1 : 2) and 30 g of the catalyst were added to the autoclave. The air was taken out from the reactor to an absolute pressure of approximately 0.005 MPa by a vacuum pump. Then, hydrogen from the cylinder was introduced into the reactor to a pressure of 0.5 MPa and maintained for 10 min to ensure that the reactor does not leak. Afterwards, H_2_ was pumped out and then raised to 0.5 MPa. This operation was repeated three times to ensure that the remaining O_2_ in the system was removed. The autoclave content was heated to the desired temperature at a stirring speed of 100 r min^−1^ and the reactor was then pressurized with hydrogen to obtain the desired partial pressure of hydrogen and then started stirring at a predetermined speed. During the reaction, the charged hydrogen was replenished from the cylinder to the reactor by manual control of the needle valve to maintain a constant hydrogen partial pressure. After the reaction was finished, the reactor content was cooled with an external electrically heated jacket until it reached approximately 100°C. The product was removed after the gas was exhausted from the reactor. C9 HPR was obtained by method pumping filtration and vacuum distillation, and the evaporated hydrogenation solvent was separately recovered and was recycled as the hydrogenation solvent used.

The bromine number of the raw materials and products were measured with a bromine valence and bromine index apparatus (denoted BVBI, Model BR-1, made in China GuoRui). The colour value of the petroleum resin was determined with a Lovibond Gardner scale 3000 comparator. The softening point was determined (Ring ball) according to ASTM D 6493-11. The C9 PR and hydrogenated products were characterized by ^1^H nuclear magnetic resonance (^1^H-NMR; Bruker NMR 400 MHz Ultra Shield). The functional groups were investigated by FTIR. Molecular weight (Mw) and molecular weight distribution (Mw/Mn) were determined by gel permeation chromatography (GPC, Waters 1525–2414, based on polystyrene standards). To reduce errors, we performed each experiment in parallel for several times. Three parallel experimental data with relative error less than 5% were regarded as final results.

## 3. Results and discussion

### 3.1. Characterization of NiO-PVP /PFC3R and NiO/PFC3R

**Figure 4.**
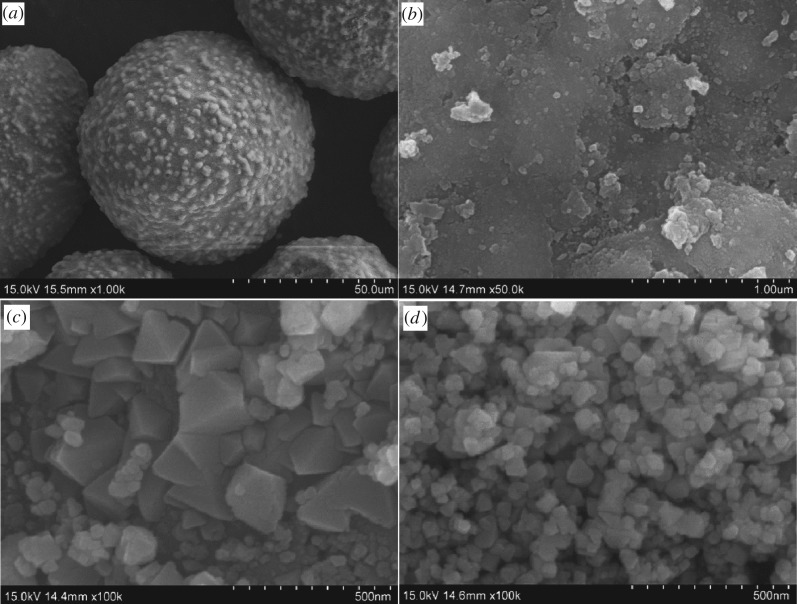
SEM micrographs of cross-sections of PFC3R (*a,b*), NiO-PVP/PFC3R (*c*) and NiO/PFC3R (*d*).

Representative SEM images of PFC3R, NiO/PFC3R and NiO-PVP/PFC3R are shown in figure 4. The PFC3R was spherical and had a rough surface wall with a porous appearance, which explained its large BET surface area. Grains of nickel oxide supported on the carrier are polyhedrons; the size of the nickel oxide grains was smaller and uniform and the dispersibility was better after the PVP was added to the catalyst.

**Figure 5.**
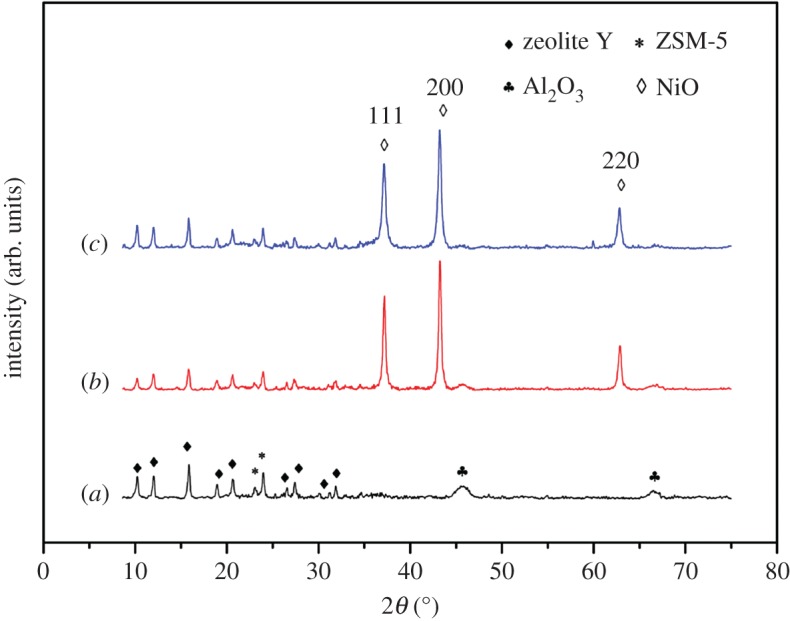
XRD patterns of PFC3R (*a*), NiO/PFC3R (*b*) and NiO-PVP/PFC3R (*c*).

The XRD patterns of the PFC3R, NiO/PFC3R and NiO-PVP/PFC3R are shown in figure 5. The PFC3R was composed of crystalline zeolite phases (mainly Y zeolite and ZSM-5 zeolite) and Al_2_O_3_, suggesting that it can be used as an excellent support material. Characteristic peaks of NiO (2*θ *= 37.3°, 43.3°, 62.8°) marked by miller indices (111), (200), (220) were observed in the 2*θ* range from 10° to 75°, which revealed that the unreduced sample was nickel oxide. The average crystallite diameter of NiO was estimated by the Scherrer formula based on the NiO (200) plane. The size of NiO in NiO/PFC3R was around 25.6 nm and that in NiO-PVP/PFC3R was 18.7 nm. The reason for such a discrepancy between the average values of the grain size in these two catalysts may be that the dispersion of PVP reduced the metal reunion.

**Figure 6.**
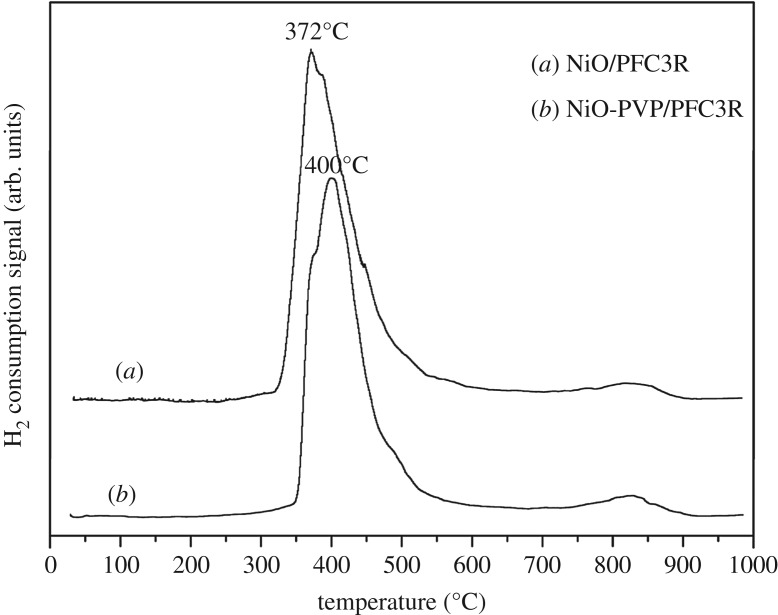
TPR profiles of NiO/PFC3R (*a*) and NiO-PVP/PFC3R (*b*).

The TPRs of NiO/PFC3R and NiO-PVP/PFC3R were conducted to characterize these catalysts (figure 6). The H_2_ consumption peaks located at 828°C were attributed to the reduction of spinel NiAl_2_O_4_. The maximum reduction peaks of NiO/PFC3R and NiO-PVP/PFC3R were present at 371°C and 403°C, respectively. A possible explanation for this difference is that the nickel oxide grains have a stronger force with the support after the addition of auxiliaries.

The H_2_-TPD experiments were performed to obtain information on the surface structure as well as to quantitatively determine the amount of chemisorbed hydrogen, which in turn is a good estimate of the metal dispersion and surface area. Hydrogen absorption properties from the H_2_-TPD measurements of NiO/PFC3R and NiO-PVP/PFC3R are listed in table 2, along with the nickel loading as determined by ICP-AES. The Ni dispersion in NiO-PVP/PFC3R was significantly higher than that in NiO/PFC3R.

**Table 2:** Hydrogen absorption properties of NiO/PFC3R and NiO-PVP/PFC3R.

sample	Ni loading (%)^a^	H_2_ uptake (ml g^−1^ STP)^b^	dispersion (%)	Ni specific surface area (m^2^ g^−1^ STP)
NiO/PFC3R	12.84	0.89	6.05	40.27
NiO-PVP/PFC3R	12.79	1.35	9.21	61.32

^a^As determined by ICP before H_2_ reduction.

^b^As determined by H_2_-TPD.

The specific area and other textural properties of the supports and catalysts are listed in table 3. The specific surface area and the pore volume of PFC3R were much larger and the average pore size was smaller after the acid treatment, probably because the support has a richer pore structure and more micropores were produced owing to the etching effect of dilute sulfuric acid. After impregnation with the nickel solution, the specific surface area and pore volume of the catalyst decreased and the average pore size increased, suggesting that the nickel oxide grains were loaded on the surface and the pores of the support. The specific surface area of Ni-PVP/PFC3R was much higher than that of Ni/PFC3R because of the smaller particle sizes and the better dispersity of Ni [29].

**Table 3.** Textural properties of FC3R, NiO/PFC3R and NiO-PVP/PFC3R.

sample	specific surface area (m^2^ g^−1^)	most probable pore size (nm)	total pore volume (cm^3^ g^−1^)
FC3R	67.97	7.10	0.10
PFC3R	124.60	5.83	0.15
NiO/PFC3R	61.03	6.63	0.08
NiO-PVP/PFC3R	83.35	6.65	0.12

**Figure 7.**
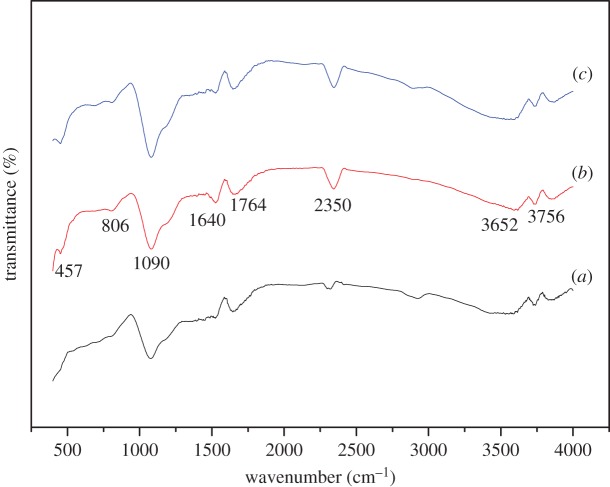
FTIR spectra of PFC3R (*a*), NiO /PFC3R (*b*) and NiO-PVP/PFC3R (*c*).

The FTIR spectra of PFC3R (*a*), NiO/PFC3R (*b*) and NiO-PVP/PFC3R (*c*) are presented in figure 7. The FTIR spectrum of these three samples is similar, which indicated that the nickel loading had no effect on the structure of PFC3R. The broad band centred at approximately 1090 cm^−1^ in the FTIR spectra of PFC3R, which has been referred as the ‘main band' in geopolymer spectra [30–32], was ascribed to the asymmetric stretching vibration of TO_4_ (T: tetrahedral Si or Al); the appearance of a peak with an absorbance at 806 cm^−1^ was attributed to the bending modes of that species. The absorbance at 2350 cm^−1^ was explained by the presence of ambient CO_2_. The bands at 3756 cm^−1^, 3652 cm^−1^ and 1640 cm^−1^ were assigned to asymmetric stretching, symmetrical stretching and bending vibrations of physically absorbed water. The vibration peaks of NO_3_^−^ at 1382 cm^−1^ and 823 cm^−1^ [33] were not observed, which suggested that the nitrates on the catalyst were completely decomposed after calcination.

**Figure 8.**
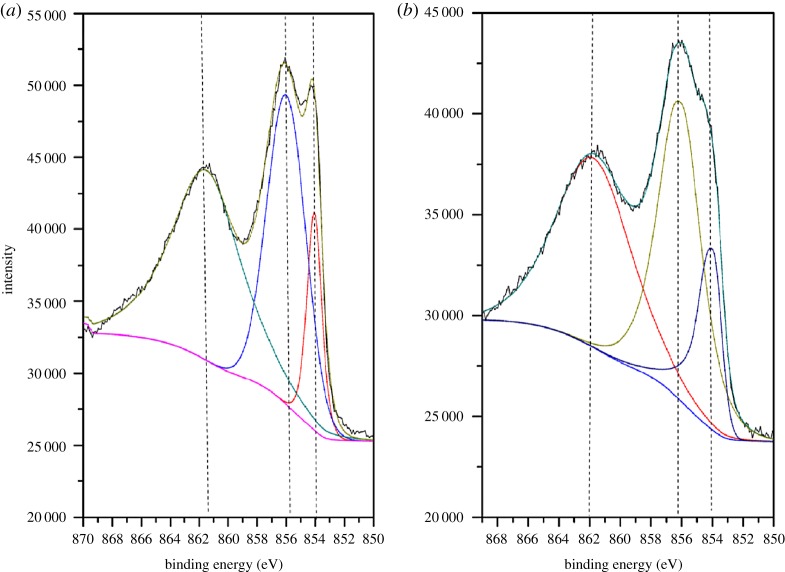
XPS spectra of Ni 2p3/2 region of NiO-PVP/PFC3R (*a*) and NiO/PFC3R (*b*).

Several XPS studies of oxidized and reduced forms of supported and unsupported nickel catalysts have been reported in the literature [34,35]. As shown in figure 8, the XPS spectra of NiO/PFC3R and NiO-PVP/PFC3R in the Ni 2p_3/2_ region were essentially identical. Both spectra contained three peaks, appearing at binding energies of approximately 854, 856 and 862 eV, suggesting that there were three different Ni sites on the support. The first peak centred at approximately 854 eV was attributed to the binding energy of NiO dispersed on the surface of the carrier [36], and the second peak centred at 856 eV was characteristic of Ni 2p_3/2_ in NiO particles interacting more strongly with the support [36]. The interaction between NiO and PFC3R in NiO-PVP/PFC3R was much stronger than that in NiO/PFC3R, which was indicated by the ratios of the area between the second and first peak of the two spectra. The peak located at approximately 862 eV was assigned to Ni^2+^ ions in a network of Ni(OH)_2_, NiAl_2_O_3_ and NiAl_2_O_4_ compounds [37].

### 3.2. Catalyst testing over Ni-PVP/PFC3R catalyst and Ni /PFC3R

#### 3.2.1. Activity of the catalysts

**Figure 9.**
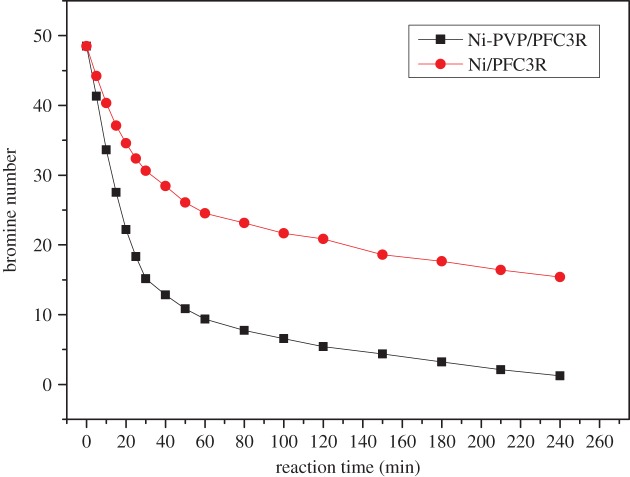
Activity of the catalysts for C9 PR hydrogenation. Reaction conditions: resin, 30% C9 solution 900 ml; catalyst, 30 g; H_2_ pressure, 8 MPa; temperature, 270°C; time 4 h.

Hydrogenations of C9 PR were performed over the Ni-PVP/PFC3R and Ni/PFC3R catalysts to evaluate the activity of both catalysts. The results are shown in figure 9. As the reaction time increases from 0 to 120 min, the bromine number of the PR over Ni-PVP/PFC3R rapidly decreased from 48.50 to 5.42; by contrast, the bromine number over Ni/PFC3R was only 20.87 at the same time. When the reaction time reached 4 h, the bromine value dropped to 1.25 g Br/100 g and the Gardner colour over Ni-PVP/PFC3R was 1; however, the bromine value and Gardner colour over Ni/PFC3R only decreased to 15.38 g Br/100 g and 5, respectively. Meanwhile, the sulfur content of the former fell to 10 mg kg^−1^ and the latter only dropped to 20 mg kg^−1^. These results suggested better hydrogenation activity and higher sulfur removal capacity of the Ni-PVP/PFC3R catalyst. This finding was ascribed to the smaller NiO particles and stronger NiO–PFC3R interaction.

#### 3.2.2. Catalyst stability testing

**Figure 10.**
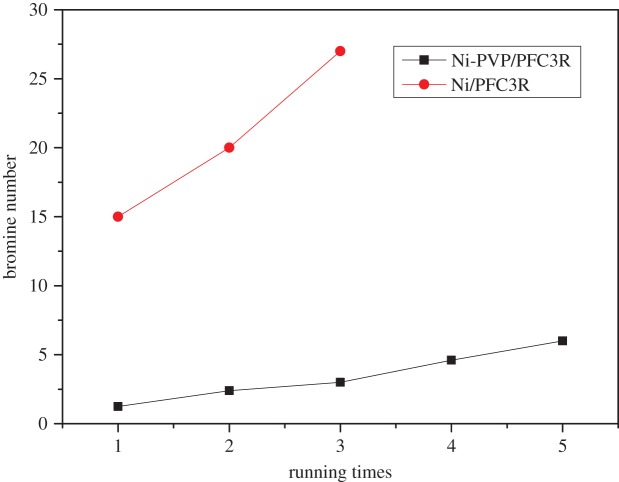
Stability of catalysts for C9 PR hydrogenation. Reaction conditions: 30% C9 solution 900 ml; catalyst, 30 g; H_2_ pressure, 8 MPa; temperature, 270°C; time 4 h.

Studies of the stability of the Ni-PVP/PFC3R and Ni/PFC3R catalysts were carried out as follows. After the first cycle of the hydrogenation was completed, the reactant was allowed to settle down until the temperature decreased to 80°C, and the supernatant product mixture was removed from the reactor. A fresh charge of reactants was added to the catalyst residue in the reactor, and a subsequent run was performed. This procedure was followed for an appropriate number of runs, and the results are shown in figure 10.

The bromine number was 6.45 g Br/100 g during the 5th cycle in the presence of the Ni-PVP/PFC3R catalyst. In comparison, the bromine number over the Ni/PFC3R catalyst increased to 26.37 after three cycles. The results indicated that the Ni-PVP/PFC3R catalyst was more stable. This was attributed to the smaller particle size and higher dispersion of nickel.

### 3.3. Effect of various factors on the performance of the Ni-PVP/PFC3R catalyst

**Figure 11.**
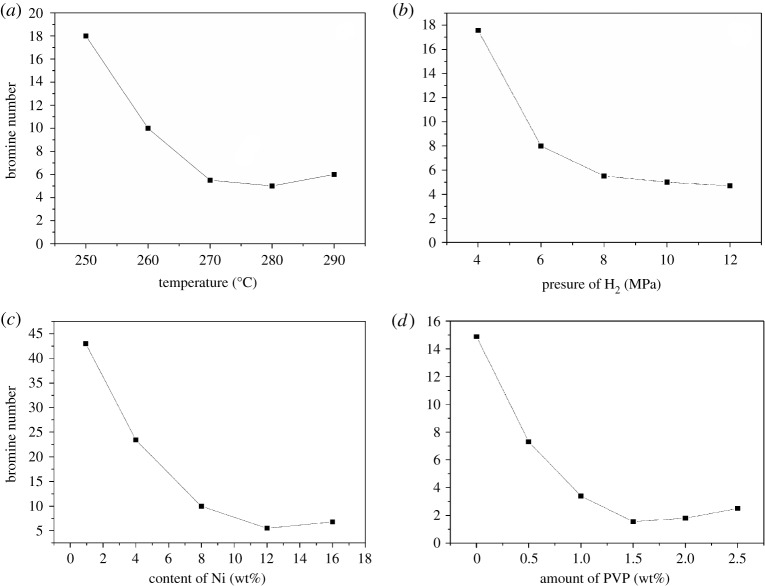
Bromine number versus various reaction conditions for Ni-PVP/PFC3R. Reaction conditions: stirring speed, 500 r min^−1^, catalyst, 30.0 g; 30% C9 solution 900 ml; (*a*) Ni loading, 12 wt%; H_2_ pressure, 8 MPa; time, 120 min, amount of PVP, 1.5 wt%; (*b*) Ni loading, 12 wt%; temperature, 270°C; time, 120 min, amount of PVP, 1.5 wt%; (*c*) H_2_ pressure, 8 MPa; temperature, 270°C, time, 120 min; amount of PVP, 1.5 wt%; (*d*) Ni loading, 12 wt%; H_2_ pressure, 8 MPa; temperature, 270°C; time, 120 min.

Figure 11*a* illustrates the effects of varying reaction temperature on the C9 PR hydrogenation. As the reaction temperatures increased from 250°C to 270°C, the bromine number rapidly decreased from 18.13 to 6.20, and when the temperature reached 280°C, the bromine number dropped to 5.66; however, as the temperature continued to increase, the bromine number increased. The initial decrease in the bromine number was explained by the improved H_2_ diffusion in resin-200^#^ oil solution with increasing temperature; however, the higher temperature could result in the degradation of C9 PR and the production of coke, which could lead to reduced activity.

The catalytic performances of Ni-PVP/PFC3R in the C9 PR hydrogenation at different H_2_ pressure at 270°C for 120 min are shown in figure 11*b*. As the H_2_ pressure increased from 4 MPa to 8 MPa, the bromine number decreases from 17.57 to 5.53; further increases in the H_2_ pressure to 10 MPa slightly decreased the bromine number to 4.78. Under a low H_2_ pressure, the concentration of hydrogen dissolved in solution is in accordance with Henry's Law. A clear increase in the reaction rate was attributed to the increase in the hydrogen concentration with increasing H_2_ pressure. The hydrogen appears to saturate at a pressure higher than 8 MPa, which leads to a smaller reduction of the bromine number.

The effect of the Ni content on reaction was investigated and the results are shown in figure 11*c*. A blank experiment over PFC3R (with a Ni loading of 0.98 wt%) was conducted; the result revealed that the bromine number decreased from 48.50 to 42.32, which indicated that the nickel metal deposited on PFC3R was active for C9 PR hydrogenation. The bromine number decreases from 23.43 to 5.51 as the Ni content increased from 4 wt% to 12 wt%, which was attributed to an increase in the availability of catalytically active sites. However, when the Ni loading was higher than 12 wt%, the bromine price was higher; one of the reasons for this result may be the partial aggregation of the active component.

Figure 11*d* shows the effect of the amount of PVP on the C9 PR hydrogenation. The bromine number decreased from 14.87 to 1.53 as the amount of PVP was increased from 0 to 1.5 wt% of the catalyst; the results indicated that the addition of PVP to the Ni catalysts resulted in better hydrogenation performance, which was attributed to the prevention of Ni particle aggregation and grain growth as a result of the steric effect of PVP. When the amount of PVP increased to 3.0 wt% of the catalyst, the bromine value slightly increased, probably because the viscosity of the solution increased, which caused a slight decrease in the catalyst loading.

### 3.4. Characterization of the petroleum resin

**Figure 12.**
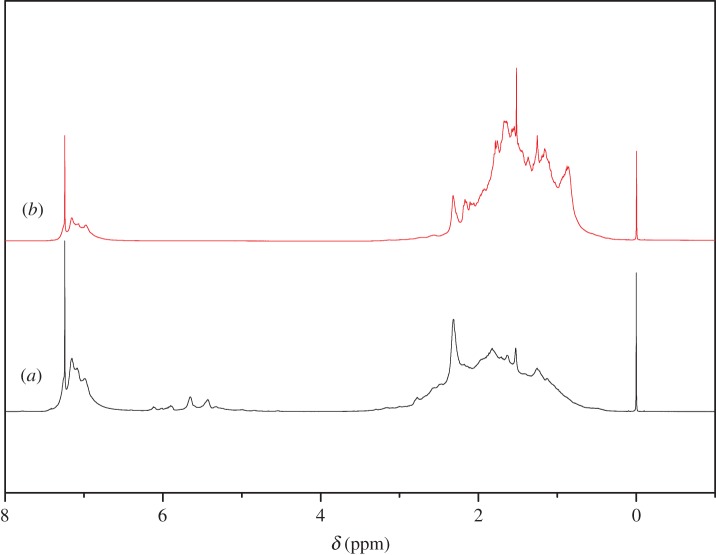
The ^1^H-NMR spectra of C9 HPR (*a*) and C9 PR (*b*).

The ^1^H-NMR spectra of C9 PR and C9 HPR are shown in figure 12. the peaks located at 6.5–7.2 ppm are assigned to aromatic proton including aromatic proton of indene and vinyl toluene unit, the corresponding peaks in the range of 0.4–3.4 ppm are proton of aliphatic proton and the peaks at 4.5–6.5 ppm represent the ethylenic C=C double bond [3]. After 4 h the hydrogenation reaction, both ethylenic unsaturated parts and aromatic unsaturated parts (the peaks at 4.5–6.5 and 6.5–7.2 ppm) decreased simultaneously and aliphatic proton parts increased, it is noted that the peak areas at 4.5–6 ppm were completely eliminated, indicating that the ethylenic unsaturated parts and aromatic proton in C9 PR were eliminated mostly, and the carbon–carbon double bond in polymer was first hydrogenated.

**Figure 13.**
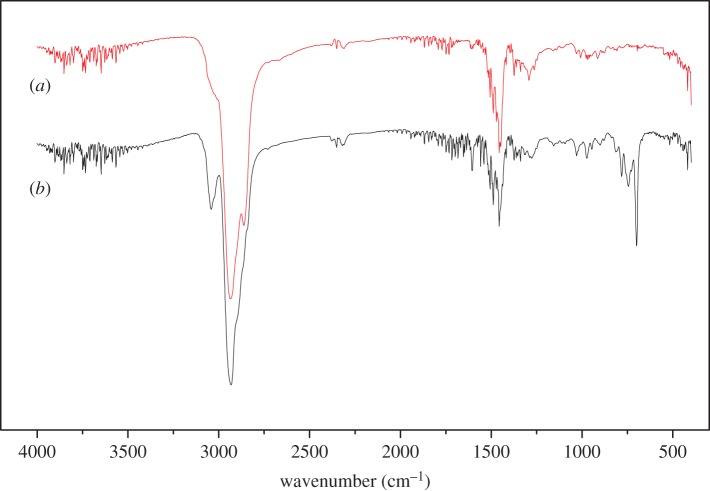
FTIR spectra of C9 HPR (*a*) and C9 PR (*b*) over Ni-PVP/PFC3R at 8 MPa and 270°C for 4 h.

The FTIR spectra of C9 PR and C9 HPR are shown in figure 13. The bands observed at 2930–2880 cm^−1^ could be assigned to stretching of the CH bond of methyl and methylene groups [38]. The CH_3_ symmetric bending and CH_3_ in-plane bending frequencies were attributed at 1373, 1364 cm^−1^ and 1487, 1453 cm^−1^, respectively [39]. The bands at 1660–1640 cm^−1^ were attributed to *ν*C=C vibration of olefin [40]. Accordingly, the results suggested that the olefin was eliminated after hydrogenation. The band at approximately 3050 cm^−1^ was attributed to the aromatic C=C stretching, whereas the bands at approximately 750 cm^−1^ and 700 cm^−1^ were assigned to aromatic C–C bending [5]. The intense decrease of these vibration peaks indicated that the aromatic hydrocarbons were mostly eliminated after hydrogenation. These results were in good agreement with the ^1^H-NMR results.

**Figure 14.**
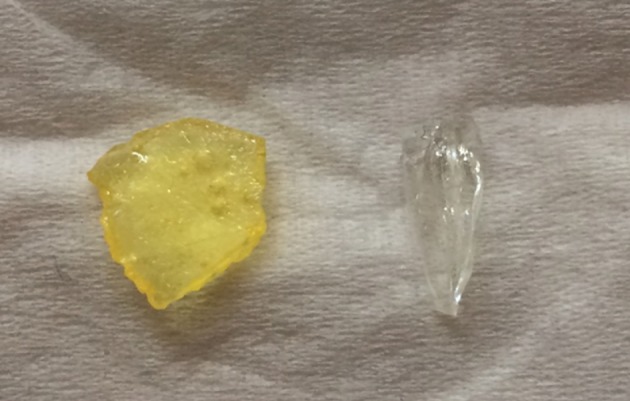
Colour reduction of the C9 PR after hydrogenation over the Ni-PVP/PFC3R catalyst.

After hydrogenation, the C9 HPR with a bromine number of 1.25 have near water to water white colour (figure 14), the softening point of the PR decreased from 126°C to 118°C, a similar phenomenon also appeared with γ-Al_2_O_3_ used as support of catalyst for C9 PR hydrogenation in Yu *et al*.' s research [4]. They found that the softening point decreased slightly by 4% after hydrogenation, while ours decreased slightly 6%, which indicated that the influence of the support on the decrease of the softening point was not significant. Meanwhile, the Mw decreased from 1725 to 1290, and the Mw/Mn became narrower in our research (table 4), which was attributed to the degradation of some resin under high temperature [4].

**Table 4:** Molecular weight and softening point of C9 PR and C9 HPR.

sample	bromine value, g Br/100 g	Mw^b^	Mw/Mn^b^	softening point (°C)^c^
C9 PR	48.50	1725	1.59	126
C9 HPR	1.25	1290	1.45	118

^a^Determined by bromine valence and bromine index apparatus measured.

^b^Determined by GPC measurement.

^c^Determined by ASTM D 6493-11.

## 4. Conclusion

A PFC3R-supported Ni catalyst with PVP as a dispersant was prepared and used for the hydrogenation of C9 PR. The XRD and SEM results showed that NiO particles were dispersed homogeneously and their size was smaller after the addition of PVP as a dispersant. The XPS and TPR measurements indicated that relatively strong metal–support interactions existed between the Ni particles and PFC3R. The catalyst exhibited superior catalytic hydrogenation performance and stability for C9 PR hydrogenation in the presence of PVP, which was attributed to the good Ni distribution, small NiO crystallite size, and interactions between NiO and PFC3R. The results indicate that PVP prevents agglomeration and crystallization of nickel. Furthermore, PFC3R, which is an industrial waste, was used as the catalyst support. Therefore, it provides significant economic and environmental benefits. The bromine value was reduced to 1.25 under the following conditions: nickel content of 12 wt%, PVP amount of 1.5 wt%, temperature of 270°C, H_2_ pressure of 8 MPa and reaction time of 240 min.

## Supplementary Material

Supplementary material

## Data accessibility

The datasets supporting this article have been uploaded as part of the electronic supplementary material.
