# Supplementary material for: A Ni-based catalyst with polyvinyl pyrrolidone as a dispersant supported in a pretreated fluid catalytic cracking catalyst residue for C9 petroleum resin (C9 PR) hydrogenation
Source: R Soc Open Sci. 2018 May 23;5(5):172052. doi: 10.1098/rsos.172052 (PMC5990735; doi:10.1098/rsos.172052)
Supplement: Supplementary material [file rsos172052supp1.docx]

**Royal Society Open Science**

**Supplementary information**

**A Ni-based catalyst with** **Polyvinyl pyrrolidone (PVP) as a dispersant supported in a pretreated fluid catalytic cracking (PFC3R) catalyst residue for C9 petroleum resin hydrogenation**

Dong Chen, Linlin Wang^*^, Xiaopeng Chen^*^, Xiaojie Wei, Jiezhen Liang, Jiao Jiang, Baofang Liang

**Table S1.** Activity of the catalysts for C9 petroleum resin hydrogenation. Reaction conditions: resin, 30% C9 solution 900 mL; catalyst, 30 g; H_2_ pressure, 8 MPa; temperature, 270 ℃; time 4 h.

| Time  (min) | Bromine number  (g Br/100 g)^a^ | Standard error  δ% | Bromine number  (g Br/100 g)^b^ | Standard error  δ% |
| --- | --- | --- | --- | --- |
| 0 | 48.50 | 3.45 | 48.50 | 4.12 |
| 5 | 41.31 | 4.23 | 44.21 | 3.54 |
| 10 | 33.63 | 3.34 | 40.34 | 2.76 |
| 15 | 27.54 | 2.23 | 37.11 | 4.18 |
| 20 | 22.21 | 3.78 | 34.58 | 3.44 |
| 25 | 18.31 | 2.34 | 32.39 | 2.69 |
| 30 | 15.17 | 1.23 | 30.62 | 1.45 |
| 40 | 12.83 | 3.21 | 28.44 | 2.98 |
| 50 | 10.85 | 2.11 | 26.08 | 3.54 |
| 60 | 9.35 | 2.78 | 24.52 | 3.33 |
| 80 | 7.75 | 4.34 | 23.15 | 4.12 |
| 100 | 6.55 | 4.23 | 21.65 | 3.43 |
| 120 | 5.42 | 4.09 | 20.87 | 4.22 |
| 150 | 4.38 | 3.17 | 18.61 | 2.34 |
| 180 | 3.23 | 3.56 | 17.64 | 4.12 |
| 210 | 2.12 | 1.34 | 16.41 | 1.68 |
| 240 | 1.25 | 3.95 | 15.38 | 3.89 |

a Ni-PVP/PFC3R HPR.

b Ni /PFC3R HPR.

**Table S2.** Stability of catalysts for C9 petroleum resin hydrogenation. Reaction conditions: 30% C9 solution 900 mL; catalyst, 30 g; H_2_ pressure, 8 MPa; temperature, 270 ℃; time 4 h.

| Running times | Bromine number  (g Br/100 g)^a^ | Standard error  δ% | Bromine number  (g Br/100 g)^b^ | Standard error  δ% |
| --- | --- | --- | --- | --- |
| 1 | 1.25 | 4.34 | 15.34 | 3.87 |
| 2 | 2.45 | 4.45 | 20.78 | 4.61 |
| 3 | 3.12 | 3.78 | 26.37 | 3.11 |
| 4 | 4.81 | 3.14 |  |  |
| 5 | 6.45 | 4.13 |  |  |

a Ni-PVP/PFC3R HPR.

b Ni /PFC3R HPR.

**Table S3.** Effects of varying reaction temperature on the C9 PR hydrogenation. Reaction conditions: stirring speed, 500 r min^−1^, catalyst, 30.0 g; 30% C9 solution 900 ml; Ni loading, 12 wt.%; H_2_ pressure, 8 MPa; time, 120 min, Amount of PVP, 1.5 wt.%.

| Temperature  (℃) | Bromine number  (g Br/100 g) | Standard error  δ% |
| --- | --- | --- |
| 250 | 18.13 | 3.44 |
| 260 | 11.13 | 2.63 |
| 270 | 6.20 | 2.54 |
| 280 | 5.66 | 3.54 |
| 290 | 6.23 | 4.11 |

**Table S4.** Effects of varying H_2_ pressure on the C9 PR hydrogenation. Reaction conditions: stirring speed, 500 r min^−1^, catalyst, 30.0 g; 30% C9 solution 900 ml; Ni loading, 12 wt.%; temperature, 270 ℃; time, 120 min, Amount of PVP, 1.5 wt.%.

| H_2_ pressure  (MPa) | Bromine number  (g Br/100 g) | Standard error  δ% |
| --- | --- | --- |
| 4 | 17.57 | 4.32 |
| 6 | 8.19 | 3.41 |
| 8 | 5.53 | 3.68 |
| 10 | 4.78 | 2.51 |
| 12 | 4.54 | 3.19 |

**Table S5.** Effects of varying Ni loading on the C9 PR hydrogenation. Bromine number versus various reaction conditions for Ni-PVP/PFC3R. Reaction conditions: stirring speed, 500 r min^−1^, catalyst, 30.0 g; 30% C9 solution 900 ml; H_2_ pressure, 8 MPa; temperature, 270 ℃, time, 120 min; Amount of PVP, 1.5 wt.%.

| Ni loading  (wt.%) | Bromine number  (g Br/100 g) | Standard error  δ% |
| --- | --- | --- |
| 0.95 | 42.32 | 3.29 |
| 4 | 23.43 | 4.16 |
| 8 | 9.95 | 3.21 |
| 12 | 5.51 | 3.78 |
| 16 | 6.78 | 4.12 |
| 20 | 9.12 | 2.34 |

**Table S6.** Effects of varying amount of PVP on the C9 PR hydrogenation. Bromine number versus various reaction conditions for Ni-PVP/PFC3R. Reaction conditions: stirring speed, 500 r min^−1^, catalyst, 30.0 g; 30% C9 solution 900 ml; Ni loading, 12 wt.%; temperature, 270 ℃; time, 120 min.

| Amount of PVP  (wt.%) | Bromine number  (g Br/100 g) | Standard error  δ% |
| --- | --- | --- |
| 0 | 14.87 | 2.14 |
| 0.5 | 7.35 | 4.56 |
| 1.0 | 3.41 | 3.45 |
| 1.5 | 1.53 | 3.82 |
| 2.0 | 1.82 | 2.23 |
| 2.5 | 2.54 | 2.64 |
